# Correspondence

**Published:** 1912-08

**Authors:** Irving M. Snow, L. Kauffman

**Affiliations:** Committee on Poliomyelitis, Buffalo Academy of Medicine; Committee on Poliomyelitis, Buffalo Academy of Medicine


### CORRESPONDENCE

---

Buffalo, N. Y., July 16, 1912.

Dear Doctor:—

A few scattered cases of *infantile paralysis* have recently occured in Buffalo and in neighboring towns. The attention of the profession is called to the necessity of immediately reporting the disease to the Health Department. It is also suggested that cases of temporary paralysis of the extremities, meningitis and neuritis of the limbs may be atypic poliomyelitis. The diseases is communicable and is often spread by so-called abortive cases simulating influenza or acute indigestion.

A prompt report to the Health Department and a strict quarantine may prevent a dangerous epidemic.

Health Commissioner Fronczak is in accord with this communication.

IRVING M. SNOW, M. D.,
L. KAUFFMAN, M. D.,
Committee on Poliomyelitis,
Buffalo Academy of Medicine.

